# Molecular analysis of the mitochondrial markers COI, 12S rDNA and 16S rDNA for six species of Iranian scorpions

**DOI:** 10.1186/s13104-021-05449-3

**Published:** 2021-02-01

**Authors:** Parisa Soltan-Alinejad, Javad Rafinejad, Farrokh Dabiri, Piero Onorati, Olle Terenius, Ali Reza Chavshin

**Affiliations:** 1 Social Determinants of Health, Research Center, Urmia University of Medical Sciences, Urmia, Iran; 2 Department of Medical Entomology and Vector Control, School of Public Health, Urmia University of Medical Sciences, Urmia, Iran; 3 Department of Medical Entomology and Vector Control, School of Public Health, Tehran University of Medical Sciences, Tehran, Iran; 4 Department of Ecology, Swedish University of Agricultural Sciences, Uppsala, Sweden; 5 Department of Cell and Molecular Biology, Uppsala University, Uppsala, Sweden

**Keywords:** mtDNA, Scorpionidae, Buthidae, Iran

## Abstract

**Objectives:**

Annually, 1.2 million humans are stung by scorpions and severely affected by their venom. Some of the scorpion species of medical importance have a similar morphology to species with low toxicity. To establish diagnostic tools for surveying scorpions, the current study was conducted to generate three mitochondrial markers, Cytochrome Oxidase I (COI gene), 12S rDNA and 16S rDNA for six species of medically important Iranian scorpions: *Androctonus crassicauda*, *Hottentotta saulcyi*, *Mesobuthus caucasicus*, *M. eupeus*, *Odontobuthus doriae*, and *Scorpio maurus*.

**Results:**

Phylogenetic analyses of the obtained sequences corroborated the morphological identification. For the first time, 12S rDNA sequences are reported from *Androctonus crassicauda, Hottentotta saulcyi*, *Mesobuthus caucasicus* and *M. eupeus* and also the 16S rDNA sequence from *Hottentotta saulcyi*. We conclude that the mitochondrial markers are useful for species determination among these medically important species of scorpions.

## Introduction

Scorpions comprise more than 1500 species of which fifty species are considered of medical importance [1]. Annually, 1.2 million people are stung by scorpions [2], and severely affected by their venom [3]. The geographical and climatic diversity in Iran provides suitable conditions for a substantial number of scorpion species; as many as 52 species have been reported from Iran, although this number is not definitive [4]. Of these, at least seven species are dangerous to humans: *Androctonus crassicauda* Olivier, 1807; *Apistobuthus pterygocercus* Finnegan, 1932; *Hottentotta saulcyi* Simon, 1880; *Hottentotta schach* Birula, 1905; *Hemiscorpius lepturus* Peters, 1861; *Mesobuthus eupeus* Koch, 1839 and *Odonthubuthus doriae* Thorell, 1876 [4]. The difficulties in identifying species based on morphological techniques [5], as well as the low number of studies [4], are two of the reasons for the uncertainty regarding the number of scorpion species existing in Iran. Using molecular markers in addition to morphological methods could be useful for a more accurate assessment of species diversity in Iran. One example where potential misidentification of scorpion species could be of importance, regards *H. saulcyi* and *Scorpio maurus*. The two species are similar in appearance, but while the venom for *H. saulcyi* has an LD_50_ value of 0.73 mg/kg and is considered a risk for humans, *S. maurus* has a venom with an LD_50_ value of 9.37 mg/kg that is considered harmless [6].

Among available molecular markers, the mitochondrial markers have shown to be useful for both species identification and phylogenetic evaluation of scorpion species. For example, analysis of intraspecific divergence between *Androctonus* scorpions using three mitochondrial markers (the COI gene, 12S rDNA and 16S rDNA), showed that these markers could explain the deep divergence sub-clades between *Androctonus amoreuxi*,* A. australis* and *A. mauretanicus* that also reflect differences in their venom production [7]. Also, COI and 16S rDNA markers have been used for investigating the evolution of *Mesobuthus gibbosus* in the Northeastern Mediterranean [8].

The current study was carried out to determine the COI, 12S rDNA and 16S rDNA sequences of six medically important Iranian scorpions and to evaluate the utility of these markers for identification in comparison to morphology.

## Main text

### Material and methods

#### Taxon sampling

Six species of medical importance were collected: *Androctonus crassicauda* Olivier, 1807; *Hottentotta saulcyi* Simon, 1880; *Mesobuthus caucasicus* Nordmann, 1840; *Mesobuthus eupeus* Koch, 1839; *Odontobuthus doriae* Thorell, 1876 and *Scorpio maurus* Ehrenberg, 1828. The samples were collected from the West Azerbaijan Province (Northwestern Iran) and the Qom Province (Central Iran; Additional files 1, 2: Tables S1, S2). The scorpions were captured at night using ultraviolet light (wavelength 366.3 nm), which causes maximum fluorescence of their epicuticles. All collected specimens were stored in 96% ethanol. Specimens were identified using the keys of Farzanpay [9] and Dehghani and Valaie [10]. From the identified samples, one sample from each species at each site was selected for molecular investigations (Additional files 1, 2: Table S1, S2) and one leg was then removed from each specimen for DNA extraction.

#### DNA extraction. COI, 12S rDNA and 16S rDNA amplification

Genomic DNA was extracted from each specimen’s leg tissue using AccuPrep® Genomic DNA Extraction kit (Bioneer, South Korea). The primers LCO1490 5′-GGTCAACAAATCATAAAGATATTGG-3′ and HCO2198 5′-TAAACTTCAGGGTGACCAAAAAATCA-3′ were used for amplification of the COI gene [11]. The primers Sc-12F 5′-AGAGTGACGGGCAATATGTG-3′ and Sc-12R 5′- CAGCGGCTGCGGTTATAC-3′ were used for amplification of 12S rDNA [12]. The primers Sc-16F 5′-CGATTTGAACTCAGATCA-3′ and Sc-16R 5′- GTGCAAAGGTAGCATAAT-3′ were used for amplification of 16S rDNA [13]. PCR conditions for amplification of all fragments were as follows: initial denaturation at 94 °C for 5 min; 30 cycles of [94 °C for 30 s, 48 °C for 30 s, 72 °C for 30 s] and a final extension at 72 °C for 7 min.

#### Phylogenetic analysis

All amplicons of the three fragments were sequenced and acquired consensus sequences were analyzed using BLAST (http://blast.ncbi.nlm.nih.gov/Blast.cgi) in order to find and include similar sequences in the phylogenetic analysis. The phylogenetic relationship of the scorpions in the current study was inferred by using the Maximum Likelihood tree building algorithm and the Tamura-Nei model [14]. Initial tree(s) for the heuristic search were obtained automatically by applying Neighbor-Join and BioNJ algorithms to a matrix of pairwise distances estimated using the Maximum Composite Likelihood (MCL) approach, and then selecting the topology with superior log likelihood value. Evolutionary analyses were conducted in MEGA X [15].

## Results and discussion

**Fig. 1:**
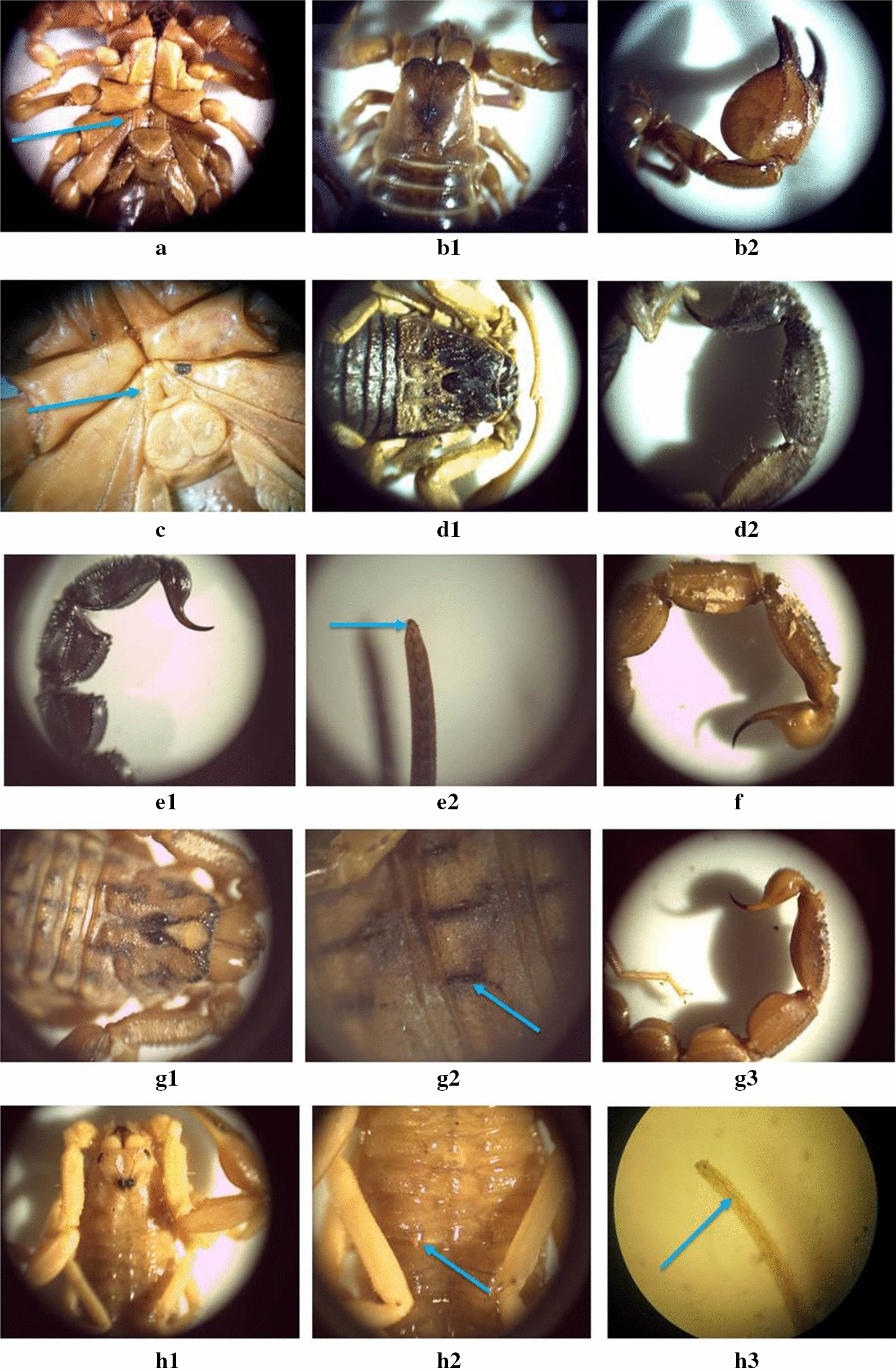
Morphological characteristics of the scorpion species of the current study. From the family Scorpionidae, which is characterized by a pentagonal sternum (**a**), is the only representative *Scorpio maurus* which is characterized by no-granule prosoma (**b1**) and a short and bulky pincer (**b2**). Species belonging to the family Buthidae have a triangular sternum as a diagnostic character (**c**). The *Hottentotta saulcyi* has a dark colored Prosoma (**d1**) and regular teeth of the abdominal region of the fifth segment of the post-abdominal and caudal vesicle (**d2**). The morphological characteristics of *Androctonus crassicauda* are the dark color of this species and the special tufts in the tail (**e1**) and, most importantly, the existence of three granules under the tooth of the fingers of the pincer (**e2**). *Odontobuthus doriae* has circular-end long teeth in the abdominal region of the tail (**f**). *Mesobuthus eupeus* has special decorations of the Prosoma (**g1**), absence of protrusion of the end pellet in the tergite (**g2**) and irregular teeth with a specific design in the abdominal region of the tail (**g3**). *Mesobuthus caucasicus* has granules along each other in carapace (**h1**), the complete protrusion of the end granule in the tergite region (**h2**), as well as the presence of an outer sub-granule being smaller than the inner sub-granule in the dental rows of the pincer (**h3**)

**Fig. 2:**
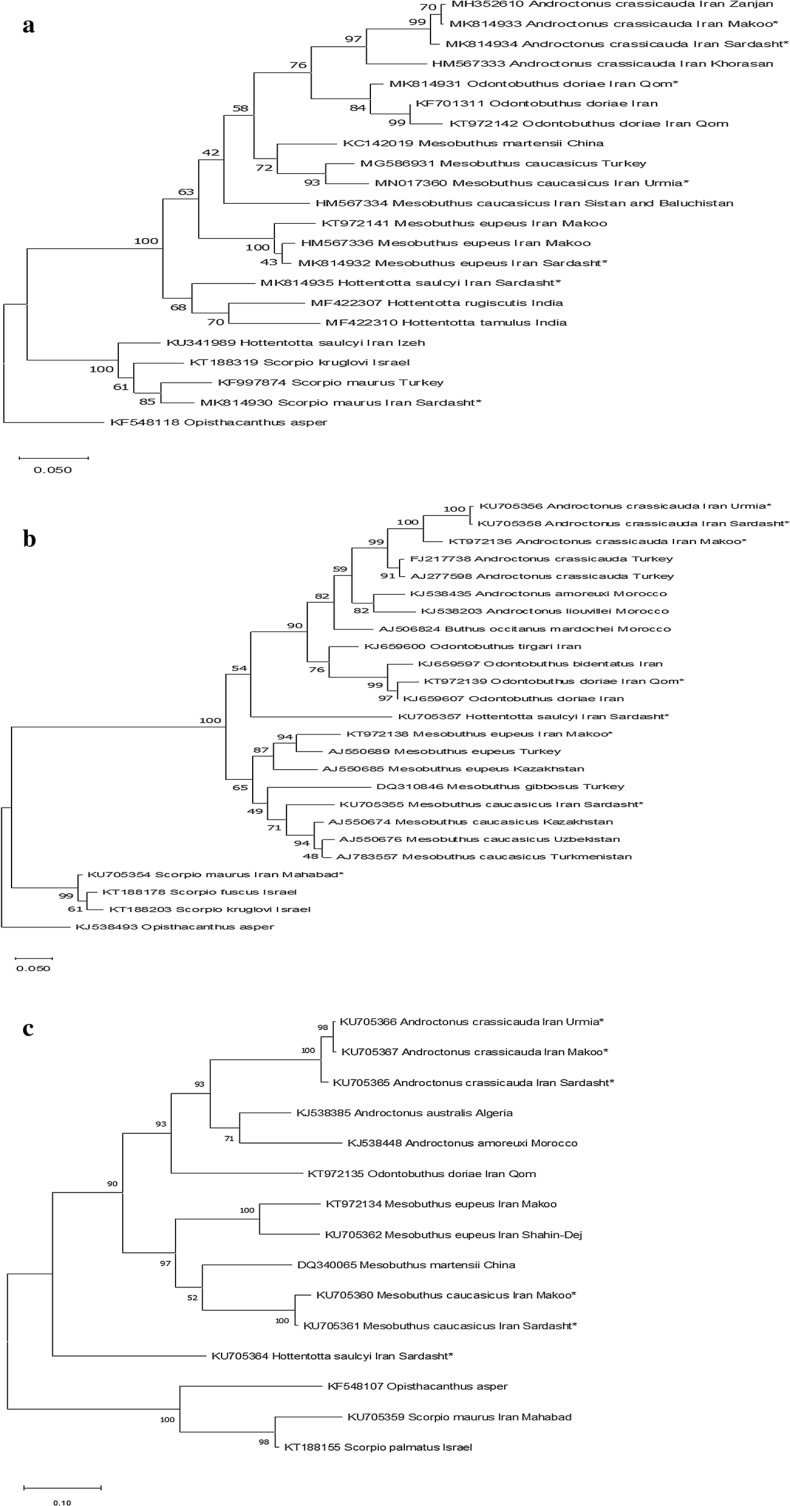
Molecular phylogenetic analysis of **a** COI, using the Maximum Likelihood method with 1000 bootstrap replications. The tree with the highest log likelihood (−4375.20) is shown. There were a total of 698 positions in the final dataset. The percentage of trees in which the associated taxa clustered together is shown next to the branches. The tree is drawn to scale, with branch lengths measured in the number of substitutions per site. Asterisks indicate sequences obtained in this project. **b** 16S rDNA, using the Maximum Likelihood method with 1000 bootstrap replications. The tree with the highest log likelihood (−3635.89) is shown. There were a total of 489 positions in the final dataset. Asterisks indicate sequences obtained in this project. **c** 12S rDNA, using the Maximum Likelihood method with 1000 bootstrap replications. The tree with the highest log likelihood (−4080.84) is shown. There were a total of 524 positions in the final dataset. Asterisks indicate sequences obtained in this project

In the current study, six species of Buthidae and Scorpionidae scorpions from northwestern and central Iran were identified based on morphological characteristics (Fig. 1). We amplified the COI gene, 12S rDNA and 16S rDNA and a representative subset was deposited in GenBank; accession numbers are shown in Additional files 1, 2: Table S1, S2 and in Fig. 2. The acquired sequences of 12S rDNA fragments in the species *Androctonus crassicauda*, *Hottentotta saulcyi*, *Mesobuthus caucasicus*, *M. eupeus* as well as 16S rDNA in *Hottentotta saulcyi* are novel. We also consider our COI sequence of *H. saulcyi* to be the first for this species, since the existing COI sequence in GenBank (KU341989) seems to be from a misidentified specimen (Fig. 2a). Additionally, the sequences of 16S rDNA of *A. crassicauda*, *M. caucasicus,* and *M. eupeus*, the 12S rDNA sequence of *Odontobuthus doriae*, and all three mitochondrial gene sequences of *Scorpio maurus* are the first reported from Iran.

In this study, we successfully used 16S rDNA to differentiate between genera, and sequences clustered well on the species level (Fig. 2b). Likewise, Cytochrome Oxidase I (COI) and 16S rDNA fragments had the appropriate capability for the differentiation of four Eurasian scorpion species (*Mesobuthus caucasicus*, *M. cyprius*, *M. eupeus*, and *M. gibbosus*; [16], which is in accordance with our results showing effective differentiation of *Mesobuthus* at the species level using all three mitochondrial markers (Fig. 2).

There has been taxonomic controversy over the placement of *Mesobuthus caucasicus*, which has been proposed to belong to the genus *Olivierius* [4, 9, 17–19] while other researchers prefer to maintain the original scientific name *Mesobuthus caucasicus* [16, 20–22]. Our phylogenies of all three mitochondrial markers strongly suggest that *Mesobuthus caucasicus* should indeed be included in the genus *Mesobuthus* (Fig. 2) and confirmed by placing *Opisthacanthus asper* as a rooted outgroup (Fig. 2).

In all, we have in this paper shown that COI, 12S rDNA and 16S rDNA are efficient markers for phylogenetic discrimination of these Iranian scorpions and set the foundations for the correct identification of these medically important species.

## Limitations

Due to the fact that the samples analyzed in this analysis were collected from limited areas, the collection of samples and species from wider areas could better indicate potential differences in morphological characteristics and genetic markers. Also, the selection of one sample of any scorpion species in each location for molecular investigation, as a limiting factor in the present study, should be considered.

## Supplementary Information

**Additional file 1: Table S1.** Summary of scorpion taxa collected, sampling localities and accession numbers of acquired 12S and 16S sequences.

**Additional file 2: Table S2.** Summary of scorpion taxa collected, sampling localities and accession numbers of acquired COI sequences.

## Data Availability

The datasets used and/or analyzed during the current study are available from the corresponding author on reasonable request.

## Abbreviations

COI: Cytochrome Oxidase 1
12S rDNA: 12S ribosomal DNA
16S rDNA: 16S ribosomal DNA
