# Supplementary material for: Molecular analysis of the mitochondrial markers COI, 12S rDNA and 16S rDNA for six species of Iranian scorpions
Source: BMC Res Notes. 2021 Feb 1;14:40. doi: 10.1186/s13104-021-05449-3 (PMC7851938; doi:10.1186/s13104-021-05449-3)
Supplement: Supplementary file 1 — Additional file 1: Table S1. Summary of scorpion taxa collected, sampling localities and accession numbers of acquired 12S and 16S sequences. [file 13104_2021_5449_MOESM1_ESM.docx]

**Table S1.** Summary of scorpion taxa collected, sampling localities and accession numbers of acquired 12S and 16S sequences.

| Species | Collection site (county) | Geographical properties | | | Accession No. | |
| --- | --- | --- | --- | --- | --- | --- |
|  |  | Longitude | Latitude | Altitude (m) | 12srRNA | 16srRNA |
| *Androctonus crassicauda* | Sardasht | 45°32'7.05"E | 36° 9'20.63"N | 994 | KU705365 | KU705358 |
| *Androctonus crassicauda* | Urmia | 44°59'0.39"E | 37°39'24.39"N | 1358 | KU705366 | KU705356 |
| *Androctonus crassicauda* | Makoo | 44°38'41.20"E | 39°43'38.95"N | 800 | KU705367 | KT972136 |
| *Hottentotta saulcyi* | Sardasht | 45°24'41.30"E | 36° 9'40.37"N | 1562 | KU705364 | KU705357 |
| *Mesobuthus caucasicus* | Sardasht | 45°32'7.05"E | 36° 9'20.63"N | 994 | KU705361 | KU705355 |
| *Mesobuthus caucasicus* | Makoo | 44°38'41.20"E | 39°43'38.95"N | 800 | KU705360 | - |
| *Mesobuthus eupeus* | Makoo | 44°38'41.20"E | 39°43'38.95"N | 800 | KT972134 | KT972138 |
| *Mesobuthus eupeus* | Shahin-Dej | 46°34'49.9"E | 36°40'48.1"N | 1350 | KU705362 | - |
| *Odontobuthus doriae* | Qom | 50°56'48.48"E | 34°37'53.14"N | 910 | KT972135 | KT972139 |
| *Scorpio maurus* | Mahabad | 45°43'3.22"E | 36°59'14.29"N | 1280 | KU705359 | KU705354 |
