# Supplementary material for: Molecular analysis of the mitochondrial markers COI, 12S rDNA and 16S rDNA for six species of Iranian scorpions
Source: BMC Res Notes. 2021 Feb 1;14:40. doi: 10.1186/s13104-021-05449-3 (PMC7851938; doi:10.1186/s13104-021-05449-3)
Supplement: Supplementary file 2 — Additional file 2: Table S2. Summary of scorpion taxa collected, sampling localities and accession numbers of acquired COI sequences. [file 13104_2021_5449_MOESM2_ESM.docx]

**Table S2.** Summary of scorpion taxa collected, sampling localities and accession numbers of acquired COI sequences.

| Species | Collection site (county) | Geographical properties | | | Accession No. |
| --- | --- | --- | --- | --- | --- |
|  |  | Longitude | Latitude | Altitude (m) | mtDNA |
| *Androctonus crassicauda* | Makoo | 44°38'41.20"E | 39°43'38.95"N | 800 | MK814933 |
| *Androctonus crassicauda* | Sardasht | 45°24'41.30"E | 36° 9'40.37"N | 1562 | MK814934 |
| *Odontobuthus doriae* | Qom | 50°56'48.48"E | 34°37'53.14"N | 910 | MK814931 |
| *Mesobuthus eupeus* | Sardasht | 45°24'41.30"E | 36° 9'40.37"N | 1562 | MK814932 |
| *Hottentotta saulcyi* | Sardasht | 45°32'7.05"E | 36° 9'20.63"N | 994 | MK814935 |
| *Scorpio maurus* | Sardasht | 45°24'41.30"E | 36° 9'40.37"N | 1562 | MK814930 |
